# Meandering conduction channels and the tunable nature of quantized charge transport

**DOI:** 10.1073/pnas.2410703121

**Published:** 2024-09-19

**Authors:** Benoit Douçot, Dmitry Kovrizhin, Roderich Moessner

**Affiliations:** ^a^Laboratoire de Physique Theorique et Hautes Energies, UMR 7589, CNRS and Sorbonne Université, Paris Cedex 05 75252, France; ^b^Laboratoire de Physique Théorique et Modélisation, CY Cergy Paris Université, CNRS, Cergy-Pontoise F-95302, France; ^c^Max-Planck-Institut für Physik komplexer Systeme, Dresden 01187, Germany

**Keywords:** Chern insulator, quantum Hall effect, edge states, Anderson localization, metrology

## Abstract

The discovery of the quantum Hall effect has established the foundation of the field of topological condensed matter physics. An amazingly accurate quantization of the Hall conductance, now enshrined in quantum metrology, is stable against any reasonable perturbation due to its topological protection. Conversely, the latter implies a form of censorship by concealing any local information from the observer. The spatial distribution of the current in a quantum Hall system is such a piece of information, which, thanks to spectacular recent advances, has now become accessible to experimental probes. It is an old question whether the original and intuitively compelling theoretical picture of the current, flowing in a narrow channel along the sample edge, is the physically correct one. Motivated by recent experiments *locally* imaging quantized current in a Chern insulator (Bi, Sb)_2_Te_3_ heterostructure [Rosen et al., *Phys. Rev. Lett.* **129**, 246602 (2022); Ferguson et al., *Nat. Mater.* **22**, 1100–1105 (2023)], we theoretically demonstrate the possibility of a broad “edge state” generically meandering away from the sample boundary deep into the bulk. Further, we show that by varying experimental parameters one can continuously tune between the regimes with narrow edge states and meandering channels, all the way to the charge transport occurring primarily within the bulk. This accounts for various features observed in, and differing between, experiments. Overall, our findings underscore the robustness of topological condensed matter physics, but also unveil the phenomenological richness, hidden until recently by the topological censorship—most of which, we believe, remains to be discovered.

## Significance

Our work addresses the question: “Where does the, famously quantized, charge current flow in a Chern insulator”. This received considerable attention in the quantum Hall effect, but the progress there was hampered by the lack of local probes, and no consensus has emerged. The fundamental problem is: topological protection is excellent at hiding local information (such as the spatial distribution of the current) – a phenomenon we call topological censorship. Two recent experiments, using local probes to determine the spatial current distribution in Chern insulator heterostructures (Bi,Sb)2Te3, have remedied the dearth of experimental data in the case of the anomalous quantum Hall effect. These experiments reached unexpected, albeit very different, conclusions. Here, we provide the theory explaining one of them.

The central pillar of topological physics is the bulk-boundary correspondence: topological properties of the gapped bulk are reflected in gapless edge states at the sample’s boundary. The standard theoretical picture of these states due to Halperin (1) encodes remarkable quantization of the Hall conductance (2–4), having a value of $e^{2}/h$ for a single chiral conducting edge channel, regardless of its length or the geometry of the sample. Deviations from this value could arise from backscattering into a counterpropagating channel, which is only available at the other edge. As long as the channels are separated by an incompressible bulk, this backscattering is suppressed, and the quantization is nearly perfect.

This picture, simple and beautiful, accounts for the experimentally observed quantization. However, it contains considerably more microscopic detail than is reflected in a single global transport coefficient. A long-standing experimental research effort conducted by the von Klitzing group (5), as well as theoretical work (6, 7), with charge transport occurring far from the edge, has indicated that, in fact, the actual situation in quantum Hall effect (QHE) systems is different from the one which is offered by the simple picture.

**Fig. 1.**
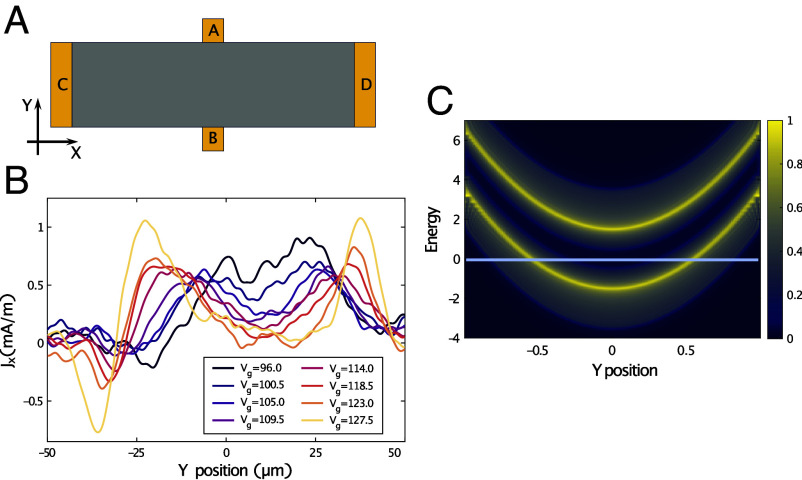
Experimental motivation and central features of Chern band. (*A*) Schematic picture of the QAHE experimental setup on the Chern insulator (Bi, Sb)_2_Te_3_ (red), where a transverse (Hall) potential $V_{\mathrm{AB}}$ between points A and B is measured upon passing a current $I_{\mathrm{CD}}$ between gold contacts C and D. (*B*) Experimental determination, ref. 11, of the spatial distribution of the quantized horizontal current along a transverse cut through the sample for (Bi, Sb)_2_Te_3_: for different gate voltages, the current can be tuned smoothly between flowing near the edge, and in the bulk of the sample. (*C*) Density of states of a disorder-free model for the Chern insulator hosting the QAHE in presence of a parabolic confining potential, with the y-axis corresponding to the transverse direction (between points A and B in Fig. 1*A*, with $V_{p}=5\times 10^{-4}$, m=−1.5; see *Materials and Methods*). The chemical potential (horizontal line) corresponds to a locally empty (near the edge), incompressible (around the center), and compressible region (in between). The meandering channel is associated with the compressible region.

Analogous quantized charge transport is also found in the quantum anomalous Hall effect (QAHE) (8), the experimental observation of which in Chern insulators, such as (Bi, Sb)_2_Te_3_ (9), has opened the field to microscopic investigations of the local current distribution, Fig. 1. Indeed, an intriguing—and technically highly impressive—pair of contemporaneous recent experiments on (Bi, Sb)_2_Te_3_ has found a bulk contribution to the current, but of apparently strikingly different nature.

One of the two experimental samples (10) suggested a rather regular flow pattern essentially dictated by the solution of Laplace’s equation in presence of a strongly anisotropic conductivity tensor; while the other (11) showed a richly structured flow pattern comprising broad channels reaching deep into the bulk. Depending on the value of the gate potential, there was a single wide conducting channel, or a pair of broad channels more or less separated by the central region with a lower current density, Fig. 1.

To account for the experimental observations in ref. 11, here we develop a theory for the QAHE in Chern insulators that assigns a prominent role to the kinetic energy (nonvanishing bandwidth) of the Chern bands. This is in sharp contrast to the physics of the QHE, where the kinetic energy in a given Landau level is quenched, and therefore Coulomb interactions and a random potential due to impurities are relatively more important in determining the spatial density profile of the electron current (5, 6, 12). The other ingredient in our model, also present in the QHE regime for 2D electronic systems, is a smooth confining potential. It is worth stressing that the disorder potential yields a quantized plateau in the QAHE, but via a different route than in the QHE.

While the complexity of the question “where the current flows” has been appreciated in the context of the QHE for a while, it is amusing to note—for all it’s worth—that artificial intelligence of ChatGPT gave us an unambiguous answer for the QAHE: “In a Chern insulator [...] the current flows along the edges of the material, rather than through its bulk.”

**Fig. 2.**
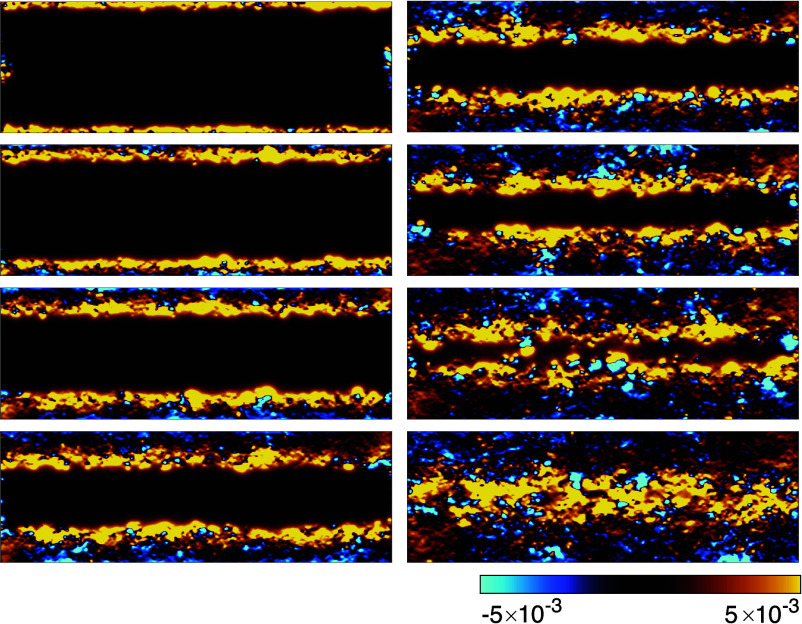
Meandering channel and its continuously variable nature. Distribution of the horizontal component of the quantized transport current (excess current generated by application of the chemical potential difference) in a Chern insulator (two-terminal Hall bar geometry). Results are shown for eight chemical potentials (μ=0.6,0.2,−0.1,−0.3,−0.4,−0.5,−0.6,−0.7, from *Top Left* to *Bottom Right*); parabolic potential $V_{p}=5\times 10^{-6}$ and disorder strength $V_{d}=1.25$ (system size =1,000×3,000 unit cells). The data are presented after applying a Lorentzian filter which mimics the spatial resolution of the experimental measurements. Corresponding charge density distributions are shown in *Materials and Methods* Fig. 8.

We theoretically predict, visually strikingly (Fig. 2), and at variance with a simple picture of edge state transport, what we call a *meandering conduction channel*, which can both be broad and spread deep into the bulk. Its width, which is typically much larger than any microscopic length, is set by the interplay of the band kinetic energy and the confining potential, with a smaller influence of disorder strength.

Note that edge states broadening in the presence of disorder was discussed in the QHE literature (13–16). However, in the case of Landau levels, this broadening requires a degree of fine-tuning as it only occurs within the narrow regions near the edges of the quantum Hall plateaux, where the 2D bulk localization length becomes comparable to the width of the Hall bar. By contrast, our mechanism produces broad meandering conduction channels in a finite range of chemical potentials, and does not require any fine-tuning.

**Fig. 3.**
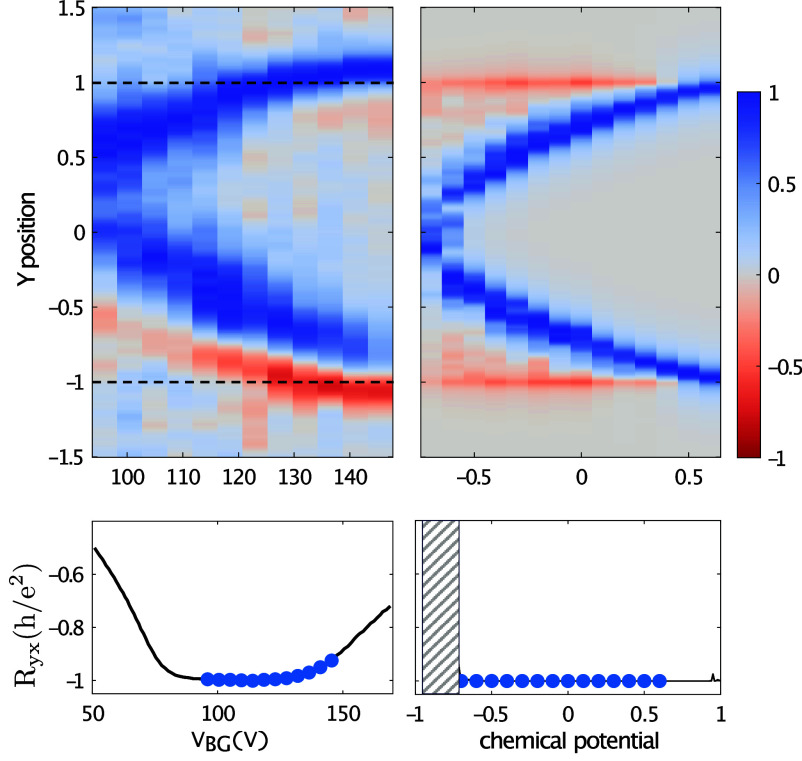
Experiment-theory comparison of the current density distribution in the QAHE. *Left*: experiment, ref. 11 (cf. Fig. 1*B*, *right*: our theory, as in Fig. 2. *Top* panels show a false color plot of the normalized current across the sample, vertical axis, against the range of backgate voltages (experiment), or chemical potential (our theory). We only show data where the current is well quantized, as indicated by the blue points in the *Bottom* panels; quantization breaks down chaotically in the shaded area. At each voltage/chemical potential, we normalize the density by the maximum of the absolute value. The vertical axis is rescaled by the half-width of the system, so that the physical sample boundaries correspond to ±1, indicated with dashed lines. For further details of comparison, see *Materials and Methods*.

Varying experimental parameters thus allows one to access a number of different regimes *all* exhibiting quantized transport. We can in particular explicitly account for the current distribution of ref. 11, as shown in Fig. 3. Our picture also resolves several questions thrown up by experiment and (apparent) theoretical dichotomies. Needless to say, the topological stability of the QAHE ensures that all of these yield the correct quantized transport coefficient, even if their detailed realizations are hugely different.

## Results

**Fig. 4.**
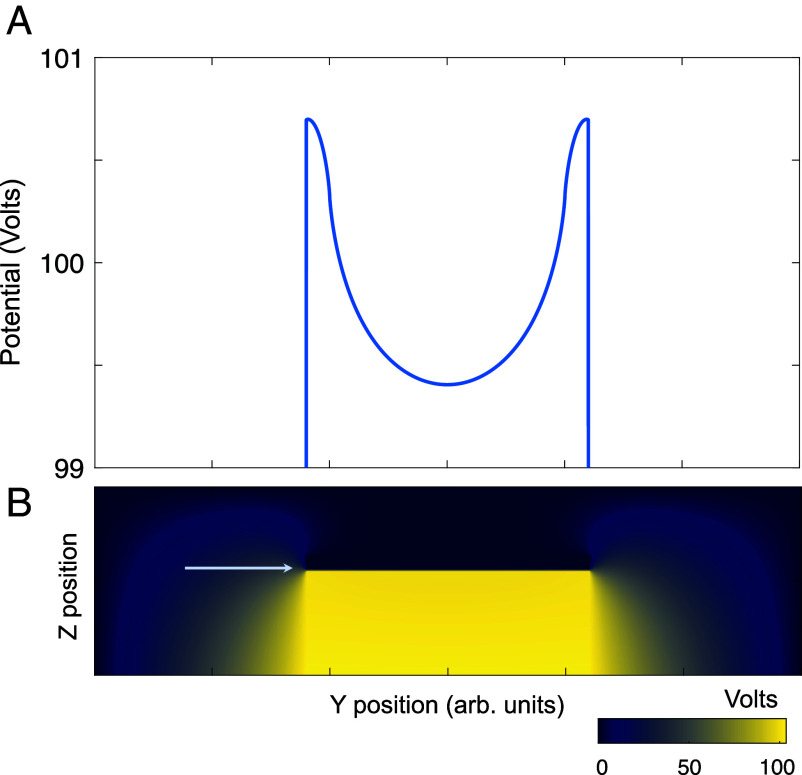
Origin of a quadratic confining potential: electrostatic potential calculated for the device geometry related to the experiment, ref. 11. (*A*) Potential profile at the position of the Chern insulator, which is separated by a thin layer of aluminum oxide from the *Top* metallic gate, and rests on a strontium titanate substrate. This is indicated by the arrow in panel (*B*), which displays a cut of the three-dimensional sample geometry transverse to its longer (x) direction. We set the voltage of the *Bottom* gate to 110 V; the dielectric constants of aluminum oxide/strontium titanate were taken as 10 and $10^{4}$ respectively, and the ratio of the width of the *Top* gate/substrate to the width of the Chern insulator was set to 1.2. Note that for this backgate voltage, we obtain a potential change at the sample location of the order of 1 V.

We have obtained spatial current distributions in a Chern insulator in a two-terminal geometry from our analysis of a standard model of the QAHE (17) in the presence of a disorder potential and a parabolic confining potential (see *Materials and Methods* Fig. 4).

**Fig. 5.**
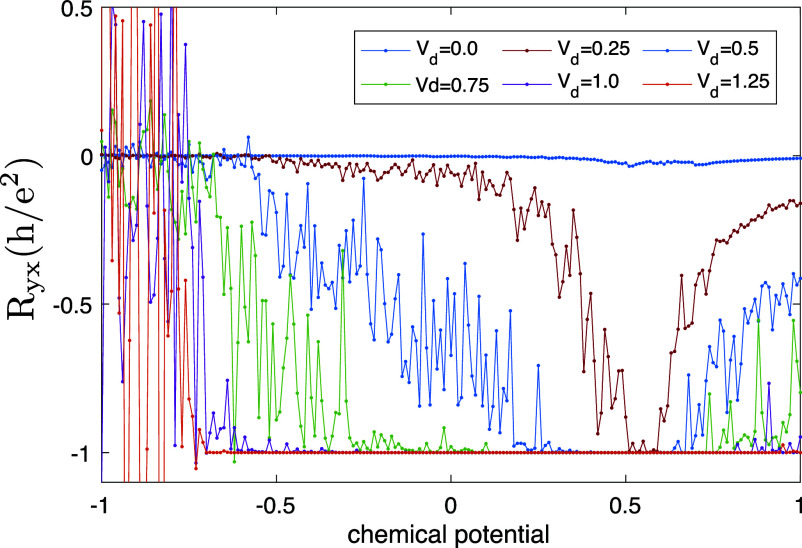
Conductance quantization in presence of disorder: Disorder is needed to yield conductance quantization over a wide range of chemical potentials. The transverse conductance $R_{\mathrm{yx}}$ was obtained by attaching two additional lateral leads to *Top* and *Bottom* of the system, e.g. in a four-terminal geometry in presence of a transverse parabolic potential with strength $V_{p}=5\times 10^{-6}$. The conductance was calculated using KWANT following the theoretical approach of ref. 22. We used a single disorder realization in the calculations for each curve (no disorder averaging), which is the reason for the large oscillations. In presence of disorder, the conductance develops a quantized plateau, which widens with increasing disorder strength, and at large values of disorder the width of the quantized plateau is of the order of the band gap in absence of disorder.

We can readily identify several strikingly different regimes in Fig. 2. First, one can see the case of a “conventional” narrow channel at each edge. These channels turn out to be continuously tunable by simply sweeping the chemical potential of the system (accessible experimentally via backgate voltage): they move into the sample and broaden in the process. Eventually, they meet in the center without any apparent spatial separation, at which point the edge of the quantization plateau is reached, and the quality of the quantization starts to degrade notably, also see *Materials and Methods*, Fig. 5.

The existence of these different regimes within a single theoretical treatment is the first central result of this work, as it accounts for the large microscopic variability of which the quantized transport is independent, and which—in the absence of local probes—is masked by the topological censorship.

### Meandering Conduction Channels.

Our second central result is implied by the first: the completely natural appearance of a broad conduction channel in the QAHE. It arises as the combination of the Chern bands of finite width separated by a gap on one hand, and the confining potential on the other, yielding a spatially inhomogeneous setup in the following way. Locally, the chemical potential can lie below both bands; within the lower (valence) band; between the bands; within the upper (conduction) band; or above both bands (Fig. 1). Most basically, the conduction properties in each of these situations are different, and hence changing the chemical potential will also change where the current flows.

Consider the horizontal line in Fig. 1*C*, where we sketched the local density of states of a disorder-free system as a function of the transverse position. Here, due to the parabolic potential, the energy at the intersection of the horizontal line with the bottom of the valence band lies within the gap between valence and conduction bands in the sample center. If one places the chemical potential at this energy, there must then be two spatially extended regions on either side of the center, where the chemical potential lies within the valence band. Quantum mechanically, these regions host equal and opposite equilibrium anomalous Hall currents (18), turning them into conducting channels with *intrinsically finite widths*. Near the boundary, no current carriers are available as the confining potential pushes the valence band above the chemical potential. At the same time, the middle region, where the chemical potential lies between the bands, is incompressible for the clean system, and can also carry current in presence of Coulomb interactions, as we discuss below. In the presence of disorder, this region hosts only localized states with a localization length of the order of a few lattice spacings.

We now turn to the discussion of the nonequilibrium current, i.e. the excess current distribution due to a chemical potential difference Δ between the lateral ends of the sample (denoted C, D in Fig. 1*A*). This leads to redistribution of charge in the two meandering channels; see *Materials and Methods*. However, while in QHE systems, we have purely chiral edge states, this is different for the QAHE, where each Chern band contains states propagating in both directions (which we refer to as propagating and counterpropagating)—rendering current quantization more delicate. How then is the quantization achieved in the Chern insulator?

### Role of Disorder.

In establishing the quantized conductance it turns out that—as in the QHE—disorder plays a crucial role; see *Materials and Methods* Fig. 5. In a nutshell, disorder Anderson-localizes all the states which are not protected by their chiral nature. This, in particular, removes the ability of all counterpropagating states to carry current. Crucially, first, the number of states which survive localization on each side of the sample (separated by the incompressible region) is given by the Chern number; and, second, their geometrical appearance and spatial distribution can vary wildly depending on disorder. Indeed, the nonequilibrium current distribution depicted in Fig. 2 (before coarse-graining) has microscopic fluctuations which are greatly in excess of the mean current densities. For the more detail-oriented reader, we emphasize with a more expansive discussion in *Materials and Methods* how differently disorder asserts itself in the QAHE in comparison with the QHE.

Generically, the current-carrying region can have the same support as the region where the chemical potential resides in the Chern band: *the appearance of a broad current-carrying region is entirely natural in this picture.* Its width is eventually determined by disorder, and primarily by a combination of the bandwidth of the Chern band, and the steepness of the potential in the region—itself externally tunable—where the chemical potential intercepts the Chern band. Our results for the four-terminal resistance are consistent with a previous analysis of the two-terminal situation in a disordered Chern insulator in a transverse electric field (19). Note that this reference considered step-like electrostatic potentials, and also found disorder-broadened conduction channels along lines where the potential jumps occur.

### Role of Coulomb Interactions.

The full disordered and interacting problem, as usual, does not offer a possibility for a theoretical analysis on the scale and detail of our above treatment of noninteracting disordered electrons. We can, nonetheless, make a number of robust qualitative observations, which turn out to be conceptually important.

In the absence of Coulomb interactions, the quantized nonequilibrium current is carried entirely by the additional electrons and holes (due to applied bias voltage) in the respective compressible strips corresponding to meandering channels. The effect of adding Coulomb interactions is then (at least) twofold—we analyze a simple illustrative phenomenological model in *Materials and Methods*, and here mention the results of importance for the following discussion of experiment.

**Fig. 6.**
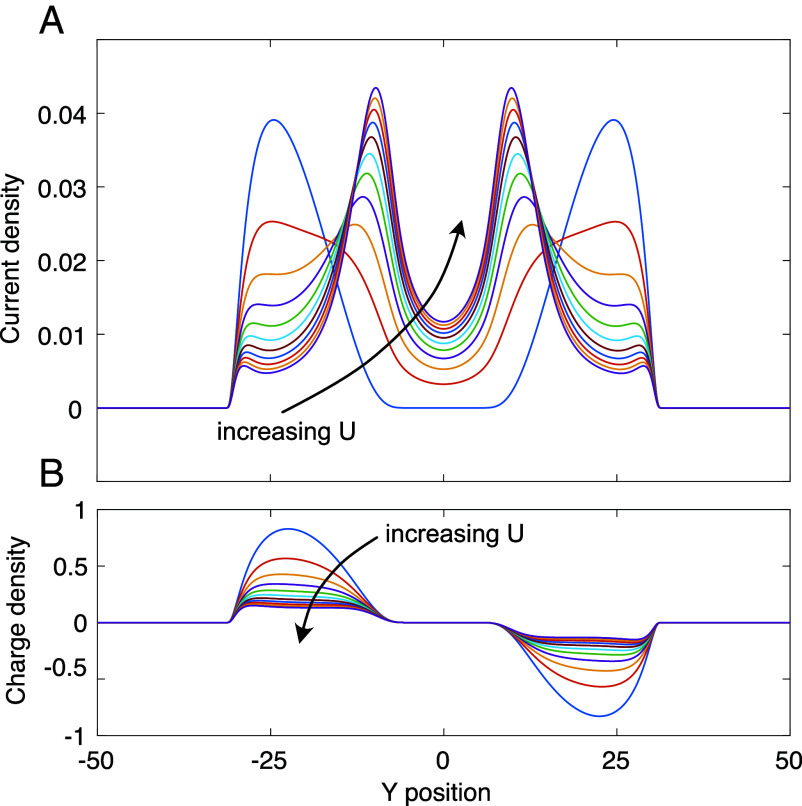
Influence of Coulomb interactions: evolution of (*A*) current and (*B*) nonequilibrium charge density: our simple phenomenological model shows that with increasing strength of the Coulomb interactions, the current distribution is moving inward within the compressible regions, and there is a simultaneous appearance of the current in the incompressible bulk around Y=0 (where the charge density remains unchanged), as the interaction strength increases from U=0 (blue line) to U=0.002 in equal steps.

First, within each channel, Fig. 6, the charge distribution flattens, while the peaks of the current distribution shift toward the center of the sample, with the inner side of the channels carrying an increasing amount of current on account of the large self-consistent electric field that develops at this position.

The second is qualitatively even more striking: the added electrons and holes in the two respective channels generate electric field in the incompressible region separating them, just like a charged capacitor with two parallel plates; thus inducing a quantized bulk current there, which is absent in the noninteracting model (U=0 vs. U>0 curves in Fig. 6). We point out that in the experimental setup (11) the current in the central incompressible region is strongly suppressed by the screening due to a nearby metallic top gate. With this in hand, we can discuss the following central question.

### Current Distribution, and Tuning between Different Regimes.

The question regarding the qualitative nature of the current distribution in the QHE or QAHE setting is often, but not always (6, 20), phrased in the form of a dichotomy: *does the current flow in the incompressible bulk or along the compressible edges ?*

Our answer to this question is twofold, and it negates the strict dichotomy: First, it depends on the detailed circumstances; and second, the current can do both at the same time. The only thing that matters for the quantization is the total electrochemical potential drop, whose detailed shape is fixed by electrostatics, disorder, and the Coulomb interactions. Only the distribution of the current is influenced in this way, not its total value. This is the topological censorship at work.

### Analysis of Experiment.

We are now in a position to connect our theoretical picture with the beautiful experimental results of refs. 10 and 11 on the QAHE in Cr-doped (Bi,Sb)_2_Te_3_ heterostructures. The Stanford experiment (10) measured the potential along the edge using local (lateral) voltage probes. It found a potential profile consistent with the current distribution arising from a translationally invariant and strongly anisotropic conductivity tensor, of the type discussed in ref. 21. This experiment seems to rule out the picture of the current flowing exclusively through a narrow edge channel, but it says little about the microscopic nature of the bulk and the conduction process through it, and indeed about the origin and the scales of the nonzero diagonal entries of the conductivity tensor. As usual, diagonal components of this tensor are associated with dissipation processes.

The Cornell experiment (11) used a micro-SQUID to measure the magnetic field distribution above the sample. From this, the current distribution was inferred for different experimental parameters, which is depicted in Figs. 1*B* and 3. The theory-experiment agreement in the latter figure is our final central result.

The most striking outcome of these measurements is the observation of a pair of broad regions in the bulk—rather reminiscent of our meandering channels—carrying the nonequilibrium current in the quantized regime. This is again at variance with the simple picture of charge transport in a narrow edge channel, while it also suggests considerably more spatial structure than the Stanford experiment. Given details of the sample geometry, preparation, gating are different between these experiments, our observation of the nonuniversality of the transport mechanism is plausibly supported by this discrepancy. The authors of ref. 11 suggest an interpretation in which this current is carried entirely in the incompressible regions. They support this claim by emphasizing that regions carrying most of the nonequilibrium current are also those in which the magnetic response to a modulation of the gate voltage reaches its maximum absolute value. The sequence of current profiles in the quantized regime is therefore interpreted as follows. The curve corresponding to the highest chemical potential on the quantized Hall plateau has two peaks, attributed to two incompressible strips. These strips gradually move toward the center of the sample as the chemical potential is reduced, and finally merge.

Based on our discussion of meandering channels, we do not see any particular reason why there should be no transport at all in the compressible regions, and at any rate, the experimental data are not precise enough to exclude this possibility. In fact, the current never seems to entirely vanish near the center of the sample, which, according to the proposed picture, is supposed to host a partially filled (hence compressible) conduction band in a finite range of chemical potentials. Also, our attempts to derive a mechanism of a purely incompressible bulk transport for realistic models, based on a Hartree–Fock treatment, were unsuccessful. For an explicit illustration of the possibility of simultaneous transport in compressible and incompressible regions, we refer to the above-mentioned Fig. 6 and discussions of the phenomenological model in *Materials and Methods*.

Our results on the quantized Hall response carried by compressible meandering channels then suggest an alternative interpretation. The large peaks are now attributed to such compressible meandering channels built from states lying inside the valence band. The residual current near the center might result from the transverse electric field across an incompressible region, due to charge imbalance between the two meandering channels, whose small value is compatible with the strong screening of Coulomb interactions due to the close proximity (∼40 nm) of the metallic top gate. When the chemical potential is reduced, the meandering channels move gradually toward the center. As they do so, they encounter a weakening of the local electric field of the confinement potential, and hence broaden.

**Fig. 7.**
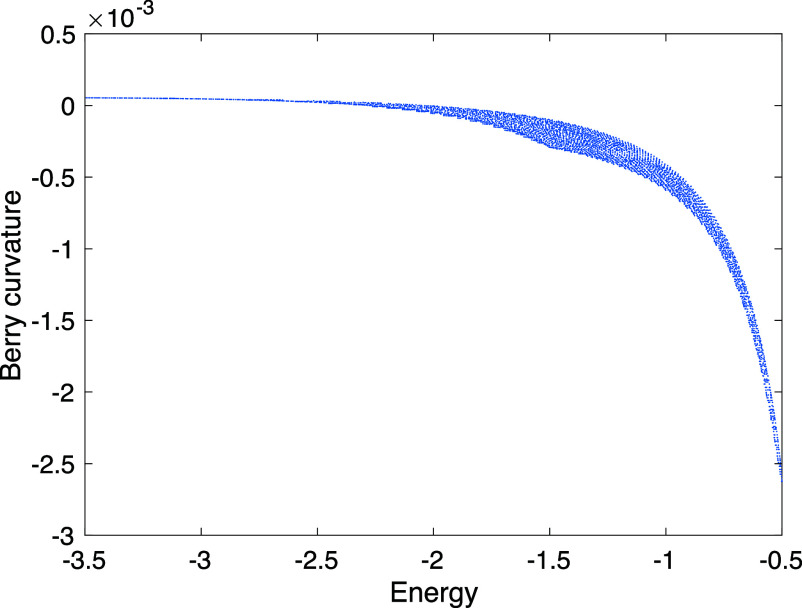
Energy dependence of Berry curvature for the model of a clean Chern insulator in the valence band: note the sign change, implying a preponderance of opposite Berry curvatures on opposite sides of the conduction channel. This figure was obtained by a scatter plot of the curvature vs. energy taken at all values of momentum in the Brillouin zone (system size 100×300 with periodic boundary conditions in both directions).

In passing, we note the asymmetry of the experimental nonequilibrium current, showing a sign-changing structure on one side, and a uniform sign on the other. This appears to involve heating, in other words, physics beyond the linear response regime (11). A sign-changing pattern is also promoted by the fact that the Berry curvature of each Chern band changes sign as a function of energy; see Fig. 7 in *Materials and Methods*. In presence of both confining potential and disorder, this translates into a tendency to carry current in opposite directions on opposite sides of the meandering channel, that appears to explain the red traces near the sample edge in the *Right* panel of Fig. 3.

## Discussion

Confronting the new microscopic experiments on the QAHE with the lore of theory of quantized Hall conductance has in our minds sharpened the following considerations. To start with, Büttiker’s theory of transport formulated in terms of conducting edge channels (22) does not per se require the existence of states which have the visual appearance of a narrow edge channel. Rather, a broad and meandering channel, resembling a stream flowing in its marshy floodplain rather than in a sterile canal, is accommodated perfectly satisfactorily. This then removes the notion that a channel meandering far from the edge into the bulk necessarily implies physics beyond Büttiker.

It does, however, not mean that ingredients such as interactions—a priori absent from the Büttiker formalism—are unimportant. But their actual role may again vary considerably in different settings. The main differences appear in the nature and physical properties of *compressible regions.* In the QHE regime, the electronic kinetic energy in a given Landau level is the same for all states. As a striking consequence, the spatial electronic distribution displays an alternation between compressible and incompressible stripes (12). For the compressible regions to be energetically stable, the local electrostatic potential has to adjust self-consistently to remain constant across them. Therefore, it is generally concluded that they carry no Hall current (5, 7, 21). By contrast, the Landau level filling factor is constant across incompressible stripes, and the electrostatic potential drop across them is responsible for almost all of the quantized Hall response, the small remaining part being carried by sharp edge states (on the scale of the magnetic length) (5, 7, 21).

In the QAHE, compressible regions created in the presence of smooth confining potentials behave very differently, in that the local electrostatic potential may now vary across them. This possibility may, at first sight, seem to imply absence of current quantization but as we demonstrated above, the current of the compressible region itself can also be quantized. We believe that this insight compels us to reconsider the status of such compressible regions in the QAHE. For instance, it is neither necessary for such regions to be absent (10), nor does the quantization preclude an electrostatic potential gradient across them (11).

Further in this vein, it is amusing to note that two popular, physically different mechanisms of charge transport, can coexist in different parts of the sample. These are, first, population imbalances due to chemical potential differences, as would occur even for charge-neutral systems such as cold atoms, and which are sufficient for narrow edge channel transport. And second, driving charged particles via forces ultimately due to an applied electric field, which would be all that is needed to account for the quantized current carried by the incompressible bulk.

Note the crucial role played by disorder also for the QAHE. It is needed to localize the counterpropagating modes in the Chern bands, which would otherwise spoil the current quantization in the compressible regions. As in the QHE, but via a different mechanism, simple disorder-free models do appear to provide the correct quantized current, but do not produce a robust plateau. While technologically challenging, a way of probing the relative relation of current flow and potential distribution would be to carry out simultaneous measurements of the potential and current distribution, i.e. by merging the experimental approaches of refs. 5 and 11. A sharp question would then be whether the current flow exists in a region without appreciable electrochemical potential drop, as would be appropriate for the meandering channel. It may also be worth noting that it will be interesting to study how local fluctuations increase with improved spatial resolution, possibly yielding insights into properties of the disorder potential.

## Outlook

We have shown how a topologically stable “universal” phenomenon of fundamental historical and conceptual importance can microscopically be realized in many, very different, “nonuniversal” ways. This is a testament to the stability of topology to deformations which may be physically striking even if they do not change the topologically fixed observables, a point perhaps somewhat obscured by the striking beauty (23) of more abstract treatments of the subject. Our results also reaffirm the crucial role played by disorder for the stability of the quantization (19), and its independence of nonuniversal features of the system.

More broadly, we believe that our work suggests that a more systematic search for different phenomenologies underpinning the same topological response is severely underexplored and likely holds many surprises. This is a most timely endeavor since recent rapid progress in the development of local probes (24–27) can now be leveraged to attack the previously inaccessible local aspects of topological systems—which may involve the appreciation of the physics taking place on various local scales. Their understanding can in turn feed into a higher degree of optimizability and controllability of designed nanosystems, such as the moiré systems which are seeing tremendous activity (28) with the recent observation of the fractional QAHE (29, 30).

## Materials and Methods

The numerical results presented in the paper were obtained using a standard theoretical model of a Chern insulator introduced in ref. 17. We consider a single-particle Hamiltonian on a square lattice with dimensions $N_{x},N_{y}$, in presence of a parabolic confining potential and on-site disorder.

The Hamiltonian describing a Chern insulator reads,

[1] $$\begin{array}{cc} {\hat{H}}_{0} & =\sum_{n}{\hat{c}}_{n}^{†}\frac{{\hat{\sigma }}_{z}-i{\hat{\sigma }}_{x}}{2}{\hat{c}}_{n+\hat{x}}+{\hat{c}}_{n}^{†}\frac{{\hat{\sigma }}_{z}-i{\hat{\sigma }}_{y}}{2}{\hat{c}}_{n+\hat{y}}+h.c.\\ & \;+m\sum_{n}{\hat{c}}_{n}^{†}{\hat{\sigma }}_{z}{\hat{c}}_{n}\;. \end{array}$$

Here, $\hat{c},{\hat{c}}^{†}$ is a fermionic annihilation (creation) operator, and ${\hat{\sigma }}_{\alpha }$ is a set of Pauli matrices. This model has been extensively studied in the case of clean systems in equilibrium (31), and it provides a good qualitative description of the BST Chern insulators studied in the above experiment. Throughout the paper, we set m=−1.5.

For the calculations of nonequilibrium behavior, such as the four-terminal conductance and current profiles, we use the KWANT package (32). All of the results are presented for lattices of dimension $N_{x}=3,000,\;N_{y}=1,000$, parabolic potential strength $V_{p}=5\times 10^{-6}$, and i.i.d. on-site disorder $V_{d}$ with a box distribution, where we used disorder strengths in the interval 0...2. The results for the nonequilibrium current and the density were obtained for a single random disorder configuration by taking a sum over 100 states in the energy window of Δ=0.02 around a given energy. For the system size used, there are about 1,000 scattering states at a given energy, so that the current is obtained from a sum containing $10^{5}$ contributions. In order to calculate the current, we apply a chemical potential difference on the two sides of the system by occupying/deoccupying scattering states on the left/right side of the sample within the energy window ±Δ/2, respectively, around the equilibrium chemical potential μ.

To make contact with experiment, the raw data for the current were postprocessed via a convolution with the Lorentzian function,

[2] $$\begin{array}{c} F\left(r\right)=F_{0}\left(a_{0}+a_{1}/\left(r^{2}+a_{1}\right)\right)e^{-\alpha r}, \end{array}$$

where the parameters $a_{0}=5,\;a_{1}=165$ were obtained by fitting the experimental measurement of the SQUID resolution, with $\alpha =2.5\times 10^{-5}$ a large-distance cutoff, and $F_{0}$ a normalization constant. It is worth noting that the current variations on the lattice scale are much larger than the average current. We also compared the results using a Gaussian filter, obtaining qualitatively similar behavior.

### Absence of the Hall Quantization for a Clean System, and the Role of Disorder in Stabilizing Conductance Plateau.

In the next few paragraphs, we present an explicit account of how the genesis of the conductance plateau differs in basic ways between the QAHE and the QHE. This is a consequence of the presence of counterpropagating states in the Chern band, which can spoil quantization in the clean case, and need to be localized by disorder. This happens as follows. Consider a situation where the chemical potential μ lies below the bottom of the valence band near the edge, and above its top near the center of the sample. In the absence of disorder, and for an infinitely long strip, translation symmetry along the x direction implies that the momentum $k_{x}$ is a good quantum number. The presence of a transverse confining potential along y yields a discrete spectrum labeled with an integer quantum number n. Let us denote by $\epsilon_{n}\left(k_{x}\right)$ the corresponding energy spectrum. In such a quasi 1D geometry, the equation $\epsilon_{n}\left(k_{x}\right)=\mu$ has a finite number of solutions $\left(n_{i},k_{n,i}\right)$, each of which defines a single conducting channel. Recall that we have two spatial intervals, on either side of the center of the strip, where the chemical potential lies within the valence band. Assuming that quantum tunneling processes between these two intervals at energy μ can be neglected, we get half of the conducting channels with spatial support lying in the upper y interval and the other half in the lower interval. Let us now concentrate on the former subset of channels. Their corresponding wave-functions are subject to a confining force directed toward the center of the strip. The simultaneous presence of this transverse force and the valence band with nonzero Chern number, Ch, creates a left–right asymmetry among these channels. Denoting by N (resp. M) the number of channels with a positive (resp. negative) group velocity $d\epsilon_{n}\left(k_{x}\right)/dk_{x}$, we have the general relation N−M=±Ch, where the sign depends on the direction of the transverse force along the y axis. This relation follows from the existence of a spectral flow $\epsilon_{n}\left(k_{x}+2\pi /a\right)=\epsilon_{n\pm \mathrm{Ch}}\left(k_{x}\right)$.

In order to evaluate the four-terminal Hall conductance of a finite clean strip, we need to specify the four-terminal transmission matrix (22). Let us label the four reservoirs by letters as indicated in Fig. 1. To simplify this discussion, we assume that these transmission matrix elements are arranged in four groups: $T_{\mathrm{CD}}≃T_{\mathrm{DC}}=\left(N+M\right)\left(1-\epsilon \right)$ (direct transmission), $T_{\mathrm{AC}}≃T_{\mathrm{DA}}≃T_{\mathrm{BD}}≃T_{\mathrm{CB}}=N\epsilon$ (chiral propagation), $T_{\mathrm{BC}}≃T_{\mathrm{DB}}≃T_{\mathrm{AD}}≃T_{\mathrm{CA}}=M\epsilon$ (chiral counterpropagation), $T_{\mathrm{AB}}≃T_{\mathrm{BA}}≃0$ (absence of direct scattering between lateral voltage probes for a large enough sample). The small parameter ϵ indicates the probability that a wave packet injected in any of the 2N propagating channels or any of the 2M counterpropagating ones will tunnel into one of the two lateral voltage probes. Because here we focus on an ideal system, we neglect in this model the contact resistances at the *Left* (C) and *Right* (D) reservoirs. This implies that all incoming wave packets from these reservoirs are eventually transmitted to other reservoirs, and this translates into $\sum_{R^{\prime }\neq R}T_{R^{\prime }R}=M+N$ for R=C,D. Following standard procedure, we obtain the Hall resistance, in units of h/e^2^,

[3] $$\begin{array}{c} R_{H}=\frac{N-M}{{\left(N+M\right)}^{2}-2MN\epsilon }. \end{array}$$

This formula is quite instructive. First, it shows that no quantization is expected in the presence of counterpropagating channels (MN≠0), because the Hall resistance depends on the value of the tunnel probability ϵ. The quantized value $R_{H}=1/N$, independent of ϵ, is recovered only in the absence of counterpropagating channels. The ubiquity of such channels for clean topological insulators in the presence of a smooth confining potential is in sharp contrast with the situation for the QHE Landau levels in a strong magnetic field, where counterpropagating edge channels are entirely absent on this level of description for a smooth confining potential.

Therefore, the role of disorder in the stabilization of sharply quantized Hall plateaus is significantly different in these two situations. For the QAHE, we have just seen that disorder is necessary to eliminate counterpropagating channels via Anderson localization, and thus to ensure a quantized Hall response carried by meandering channels. In the QHE, the clean system exhibits perfectly quantized Hall conductance as long as the chemical potential lies between two consecutive Landau levels. For a fixed electronic density, this occurs only for a discrete set of magnetic field values, corresponding to an integer number of filled Landau levels. Therefore the quantized Hall plateaus have a vanishing width, as a function of external magnetic field for a clean system. The main role of disorder in the QHE, as has been realized for a long time, is to generate plateaux of a finite width by allowing the chemical potential to change smoothly inside the gaps as the magnetic field is varied.

### Disorder-Induced Broadening of QHE Edge States.

As stated in the introduction, a disorder-based mechanism by which edge states in two-dimensional noninteracting electron systems in a strong magnetic field can spread deep inside the system bulk has been known for a long time (13–16). For such infinite systems, all states are localized, except at energies close to the centers of disorder-broadened Landau levels, corresponding to phase transitions between quantum Hall plateaus. For finite strips, almost extended edge states develop at most energies, except near plateau transitions, where the bulk localization length becomes comparable to the system width. When this happens, it has been shown that edge states spread into the bulk, thus forming broad conduction channels, as shown for example in figure 6C in ref. 16. It is however quite clear on this figure that such broad edge states exist only in a rather small energy range around the plateau transitions. By contrast, as we have shown, broad meandering conduction channels exist in disordered Chern insulators in the presence of a smooth confining potential for the whole energy range corresponding to a quantum Hall plateau. Anderson localization transitions in the same model as the one studied in this paper have been investigated in refs. 33 and 34. For moderate disorder, the phase diagram in the middle of figure 1 in ref. 33 shows that the Anderson transitions between Chern insulators and topologically trivial phases occur at energies inside the two bands of the clean system. This suggests that, as we have checked numerically in the absence of the smooth confining potential, the width of edge states remains very small, of the order of a few lattice spacings, when the energy lies inside the gap of the clean system, provided the disorder remains below the critical value where only the topologically trivial phase remains. This is also consistent with the conclusion of ref. 34. While the basic physics involved in the formation of broad QHE edge states and meandering conducting channels in Chern insulators may lead to the same universality class regarding Anderson localization transitions, we again emphasize that the combination of a wide band kinetic energy in the Chern band and of a smooth confining potential enhances the energy range in which such states exist in comparison to disorder-broadened Landau levels. This obviates the need to fine-tune the chemical potential near a plateau transition for their observation.

### Origin of the Confinement Potential.

The confinement potential, in particular its interplay with the finite bandwidth of the Chern insulator, plays an important role for the current distribution. In order to show how a parabolic potential can appear in an electrostatic problem, and to estimate its size, we have calculated the electrostatic potential in a model of the experimental system using the finite element method. *Materials and Methods* Fig. 4 displays the results of this calculation, which yields a quadratic confining potential of the form utilized in our modeling. The difference between its maximum and minimum turns out to be of the order 1 eV for a backgate voltage of 100 V. We note that, depending on the device configuration, one can obtain either sign of the parabola.

### Phenomenological Model for the Effect of Interactions on the Density and Current Distributions.

**Fig. 8.**
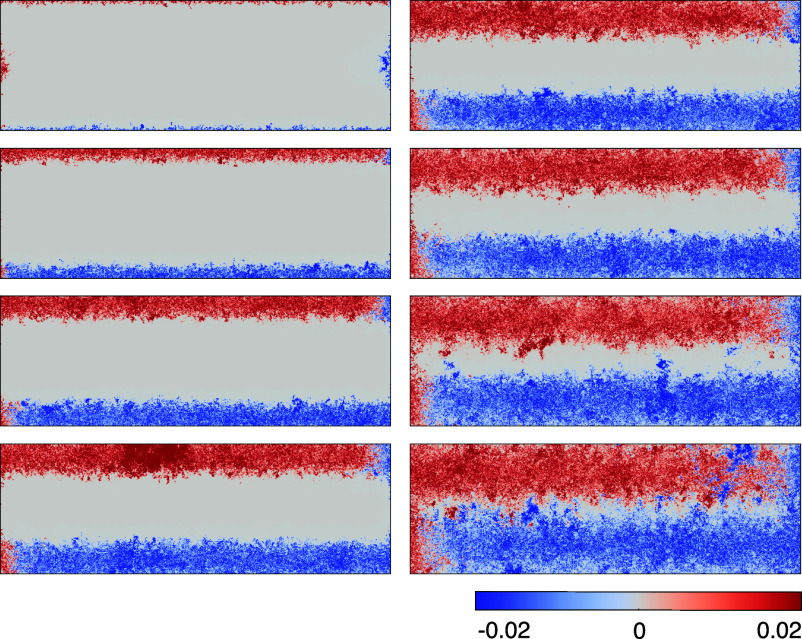
Nonequilibrium particle density (excess density) generated by the application of a chemical potential difference to the *Left*/*Right* lead of the system corresponding to and for the same parameters as the panels in Fig. 2. This shows the spatial distribution of particles/holes injected from the leads. At large chemical potentials, only the states within the narrow regions of the edge carry excess density. These compressible regions become progressively wider as we decrease the chemical potential, and merge when the latter is tuned close to the *Top* of the valence band.

Our above interpretation of current profiles in ref. 11 is based on a simple phenomenological model. Without Coulomb interactions, the quantized nonequilibrium current is carried by additional electrons and holes in the respective compressible strips hosting the meandering channels, as shown in Fig. 8. With Coulomb interactions, this pair of oppositely charged strips creates an electrostatic field across the central incompressible region, leading to an additional drop $\delta V_{I}$ in the electrochemical potential between the two strips. A total current $I_{I}$ then also flows in this central incompressible region, characterized by the same quantized Hall conductance as the meandering channels.

Based on this picture, we introduce an effective model, in which we assume translation invariance along the wire (obtained for example after averaging over disorder). Let us denote by ϕ(y) the local electrostatic potential seen by electrons, the sum of the equilibrium potential $\phi_{0}\left(y\right)$ and the induced potential, $\phi_{\mathrm{ind}}\left(y\right)$ created by the charged compressible strips, that appears under finite dc voltage bias. We assume that the particle density at energy E is a function of the kinetic energy in the valence band, which is given by E+eϕ(y). Specifically, $n\left(y,E\right)=f\left(E+e\phi \left(y\right)\right)/a_{x}a_{y}$, where f is a positive function with unit total weight, and support lying in the [0,W] interval, W being the energy width of the valence band, and $a_{x}$ and $a_{y}$ are the lattice constants along x and y directions. A second key assumption is that the local current density j(y,E) carried by a state at energy E is given by $j\left(y,E\right)=\left(e^{2}/h\right)f\left(E+e\phi \left(y\right)\right)\phi^{\prime }\left(y\right)$. In equilibrium, local particle density n(y), and current density j(y), are obtained by integrating the energy-resolved functions n(y,E) and j(y,E) over E up to the equilibrium chemical potential μ. The incompressible regions are defined within the regions which are obtained from the equation $\mu +e\phi_{0}\left(y\right)>W$, and the corresponding local density equals to $n_{0}\left(y\right)=1/a_{x}a_{y}$ in these regions, as expected for a filled valence band.

In the presence of a finite voltage bias, we integrate over energies up to $\mu_{b}=\mu +\delta \mu$ for y corresponding to the bottom half of the strip, and to $\mu_{t}=\mu -\delta \mu$ for y in the top half. This model gives a quantized Hall response for the total current I, obtained by integrating j(y) over the whole width of the sample, as $I=\left(e/h\right)\left(\mu_{b}-\mu_{t}\right)$, or equivalently $I=\left(e^{2}/h\right)\left(\phi_{t}-\phi_{b}\right)$ in terms of the total electrochemical potential difference across the sample. We denote by δn(y) and δj(y) the change in local particle and current densities after introducing the bias δμ.

The induced electrostatic potential $\phi_{\mathrm{ind}}\left(y\right)$ reads

[4] $$\begin{array}{c} \phi_{\mathrm{ind}}\left(y\right)=-\frac{e}{2\pi \epsilon_{0}}\int dy^{\prime }\;\delta n\left(y^{\prime }\right)\log\frac{\sqrt{{\left(y-y^{\prime }\right)}^{2}+d^{2}}}{|y-y^{\prime }|}, \end{array}$$

where we assume an infinitely long strip, and the presence of a perfectly conducting top electrode located at distance d above the sample. In the Cornell experiment d=40 nm. To the first order in δμ,

[5] $$\begin{array}{c} \delta n\left(y\right)=\frac{f(\mu +e\phi_{0}\left(y\right))}{a_{x}a_{y}}\left[\left(\theta \left(-y\right)-\theta \left(y\right)\right)\delta \mu +e\phi_{\mathrm{ind}}\left(y\right)\right]. \end{array}$$

To solve the coupled Eqs. **4** and **5**, one may eliminate either δn(y) or $\phi_{\mathrm{ind}}\left(y\right)$ from one of these equations, yielding an inhomogeneous linear integral equation for the remaining variable, which we have solved numerically. After this step, the nonequilibrium current in the incompressible region can be obtained from the equation

[6] $$\begin{array}{c} \delta j\left(y\right)=\frac{e^{2}}{h}\phi_{\mathrm{ind}}^{\prime }\left(y\right), \end{array}$$

whereas in the lateral compressible strips

[7] $$\begin{array}{cc} \delta j(y) & =\frac{e^{2}}{h}f\left(\mu +e\phi_{0}\left(y\right)\right)\left[\left(\theta \left(-y\right)-\theta \left(y\right)\right)\delta \mu +e\phi_{\mathrm{ind}}\left(y\right)\right]\phi_{0}^{\prime }\left(y\right)\\ & \;+\frac{e^{2}}{h}\phi_{\mathrm{ind}}^{\prime }\left(y\right)\int_{0}^{\mu +e\phi_{0}\left(y\right)}f\left(E\right)dE. \end{array}$$

The physical interpretation of this expression is quite simple. The first term arises from the change in the local electron density in the nonequilibrium state as compared to the equilibrium situation, in the presence of the *un*perturbed confining potential and hence unperturbed local anomalous velocity field. The second term may be regarded as the response (in the spirit of the Kubo formula) of a locally partially filled (compressible strips) or completely filled (incompressible strips) valence band to the additional electrostatic potential created by the change in the charge distribution.

To estimate the strength of the Coulomb interaction, we first neglect $\phi_{\mathrm{ind}}\left(y\right)$ in Eq. **5**, and compute the maximal absolute value κ(μ) of the ratio $e\phi_{\mathrm{ind}}\left(y\right)/\delta \mu$. It is reached when y lies near the centers of the compressible strips. Assuming that they are much larger than the distance d from the top gate, we estimate this ratio to be of the order of $4\left(R_{y}/W\right)\left(a_{0}d/a_{x}a_{y}\right)$, where $R_{y}$ is the Rydberg energy and $a_{0}$ is the Bohr radius. We get a rather large number (on the order of $10^{3}$) for the Cornell experiment.

Coulomb interactions modify the current profile. Most notable is a reduction of the nonequilibrium charge density δn(y) for a given δμ because the Coulomb repulsion among particles on a given compressible strip increases their total potential energy. Assuming for simplicity that only the overall scale of δn(y) is modified, but not its shape, this can be captured by a renormalization of the bias δμ into a much smaller $\delta \mu^{*}$, defined by $\delta \mu^{*}=\delta \mu -\kappa \left(\mu \right)\delta \mu^{*}$, so $\delta \mu^{*}=\delta \mu /\left(1+\kappa \left(\mu \right)\right)$. The new maximal value of $e\phi_{\mathrm{ind}}\left(y\right)/\delta \mu$ is now equal to $\kappa {\left(\mu \right)}_{\mathrm{eff}}=\kappa \left(\mu \right)/\left(1+\kappa \left(\mu \right)\right)$, which is very close to, but smaller than 1, in the physically relevant case where κ(μ) is large. Note that κ(μ) is linear in the strength $R_{y}/W$ of the Coulomb interaction. So $\delta \mu^{*}$ decreases as $W/R_{y}$ when interactions are large, but the $e\phi_{\mathrm{ind}}\left(y\right)/\delta \mu$ ratio scales to a finite limit value at large $R_{y}/W$. This value depends mostly on geometric parameters such as the widths of the compressible strips, their separation, and the effective range d of the screened Coulomb potential.

The reduction of the nonequilibrium charge density is not uniform across the compressible strips, so that δn(y) becomes more uniform, and hence effectively wider, than the noninteracting envelope function $f(\mu +e\phi_{0}\left(y\right))$. These effects are clearly seen in the numerical results shown in Fig. 6.

## Data Availability

All study data are included in the main text.
